# Double umbilical artery converging into a single umbilical artery: A case report

**DOI:** 10.1097/MD.0000000000040199

**Published:** 2024-10-18

**Authors:** Chunfang Yang, Xiaolu Yang, Lihua Qiu, Zhihui Liu

**Affiliations:** a Department of Ultrasound, The Second People’s Hospital of Yibin, Sichuan Province, Yibin, China; b School of Medical and Life Sciences, Chengdu University of Traditional Chinese Medicine, Sichuan Province, Chengdu, China; c Medical Imaging Center, The Second People’s Hospital of Yibin, Sichuan Province, Yibin, China.

**Keywords:** case report, fetus, ultrasound, umbilical arteries

## Abstract

**Rationale:**

The normal structure and Doppler parameters of the umbilical cord are closely related to many diseases, including fetal infection, chromosomal abnormalities, hypoxia, and growth and development restrictions. We report a case of bilateral umbilical artery confluence resulting in the formation of a single umbilical artery in the free segment of the fetal umbilical cord, diagnosed at 24 weeks and 4 days gestation. The fetus was born prematurely after premature membrane rupture at 31 weeks and 3 days gestation. The Toxoplasma, Others, Rubellavirus, Cytomegalovirus, Herpesvirus test showed positive results for *Toxoplasma gondii*, rubella virus, and herpes simplex virus IgG antibodies.

**Patient concerns:**

A 36-year-old woman had vaginal discharge for > 1 hour at 31 weeks + 3 days gestation and came to our obstetrics department for treatment.

**Diagnosis:**

The pregnant woman sought treatment due to premature membrane rupture and vaginal discharge for > 1 hour. The vaginal discharge was caused by *Escherichia coli*. After cesarean section, the Toxoplasma, Others, Rubellavirus, Cytomegalovirus, Herpesvirus test revealed positive results for the following: *T gondii*, rubella virus, and herpes simplex virus IgG antibodies. The patient underwent 2 ultrasound examinations and was diagnosed with umbilical artery malformation (the free segment of the umbilical cord on the fetal side converged into a single umbilical artery), which may have been related to fetal infection.

**Interventions:**

The patient received anti-inflammatory and fetal lung maturation treatment for 2 days before undergoing a cesarean section.

**Outcomes:**

The mother and newborn received anti-inflammatory, symptomatic, and supportive treatment and were discharged after 1 week of improvement. After 1 month, 6 months, and 1 year of follow-up after birth, the growth and development of the infant (height and weight) were significantly lower than those of her peers, and her responses to sound and light were slightly delayed.

**Lessons:**

Umbilical artery malformation is extremely rare and may be related to intrauterine parasitic and viral infections. Ultrasound has the advantages of being noninvasive and cost-effective and can be used to dynamically observe umbilical artery structure. An abnormal change in umbilical artery structure found during ultrasound examination can indicate intrauterine infection risk, which provides clinical guidance for further examination of pregnant women, early diagnosis, timely targeted treatment, and fetal prognosis improvement.

## 1. Introduction

Numerous permanent sequelae can result from infections caused by bacteria, rubella virus, herpes simplex virus, or *Toxoplasma* during pregnancy, including neurological defects, fetal growth restriction, and hearing loss.^[1]^ The umbilical cord, serving as the sole connection between a mother and child, experiences a sequence of pathological alterations after fetal bacterial and viral infections. Among these changes, the umbilical artery wall sustains damage and ruptures; adhesion and fusion processes then lead to the formation of a distinctive variant known as a single umbilical artery. This variant is fundamentally different from single umbilical arteries caused by abnormal embryonic development. Not only does the umbilical artery supply oxygen and nutrients to the fetus, but it also acts as a transport channel for bacteria and viruses. Fetal growth and development can be adversely affected by changes in umbilical artery structure or the transport of harmful substances, leading to increased risks of fetal growth restriction and preterm birth. This study presents a rare case report of combined intrauterine bacterial and viral infections that led to the convergence of 2 umbilical arteries into a single umbilical artery at the free segment of the umbilical cord on the fetal side. Due to the premature rupture of the membranes at 31 weeks and 3 days gestation, the weight of the newborn was 13.8th percentile (−1.1 standard deviation). Follow-up at 1 month, 6 months, and 1 year after birth revealed that the height and weight of the infant were significantly lower than those of other babies the same age, and her responses to sound and light were slightly delayed. The above situation, as well as changes in umbilical cord structure, may be related to intrauterine infection with *Toxoplasma gondii*, herpes virus, and rubella virus.

## 2. Case presentation

### 2.1. Chief complaints

The woman was 31 weeks and 3 days pregnant, and she had experienced vaginal discharge for > 1 hour.

### 2.2. History of present illness

The pregnant woman came to our hospital for prenatal systematic ultrasound screening at 24 weeks and 3 days of pregnancy. A 36-year-old pregnant woman at 31 weeks and 3 days gestation experienced vaginal discharge for > 1 hour.

### 2.3. History of previous illness

At 24 weeks and 3 days gestation, ultrasound examination at our hospital revealed structural abnormalities in the fetal umbilical artery (the free segment of the umbilical cord near the fetus converged into a single umbilical artery).

### 2.4. Personal and family history

The woman had 1 child (8 years old) that was delivered vaginally.

### 2.5. Physical examination

Light yellow, clear liquid flowed out of the vagina, and no blood was present. The cervix was already prepared for delivery.

### 2.6. Laboratory examinations

**Figure 1.**
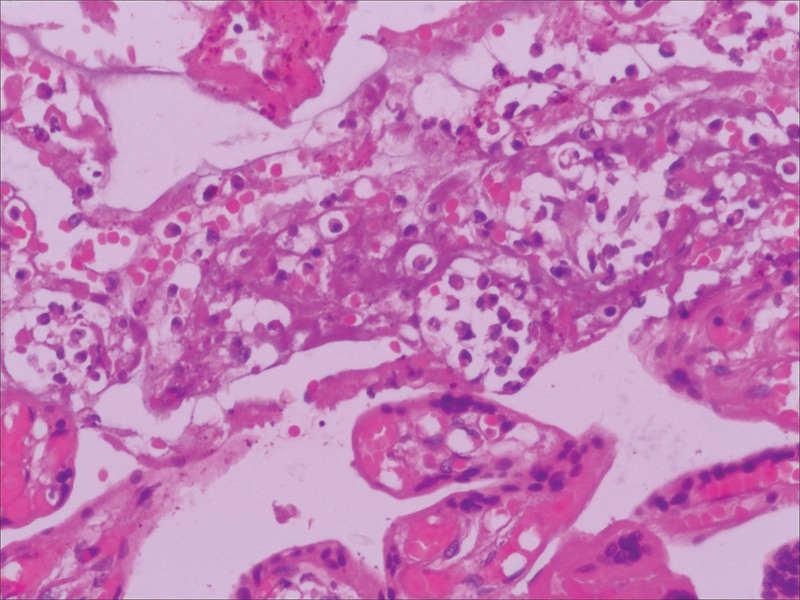
Many neutrophils are shown on a histopathological slide of placental pathology. Hematoxylin and eosin: magnification × 100.

The pathological results regarding the pregnant woman’s vaginal discharge revealed that the vaginal discharge was composed of amniotic fluid, and *Escherichia coli* was detected. The Toxoplasma, Others, Rubellavirus, Cytomegalovirus, Herpesvirus test showed positive results for *T gondii*, rubella virus, and herpes simplex virus IgG antibodies. Pathological examination of the placenta, fetal membranes, and umbilical cord revealed significant neutrophil infiltration (Fig. 1).

### 2.7. Imaging examinations

**Figure 2.**
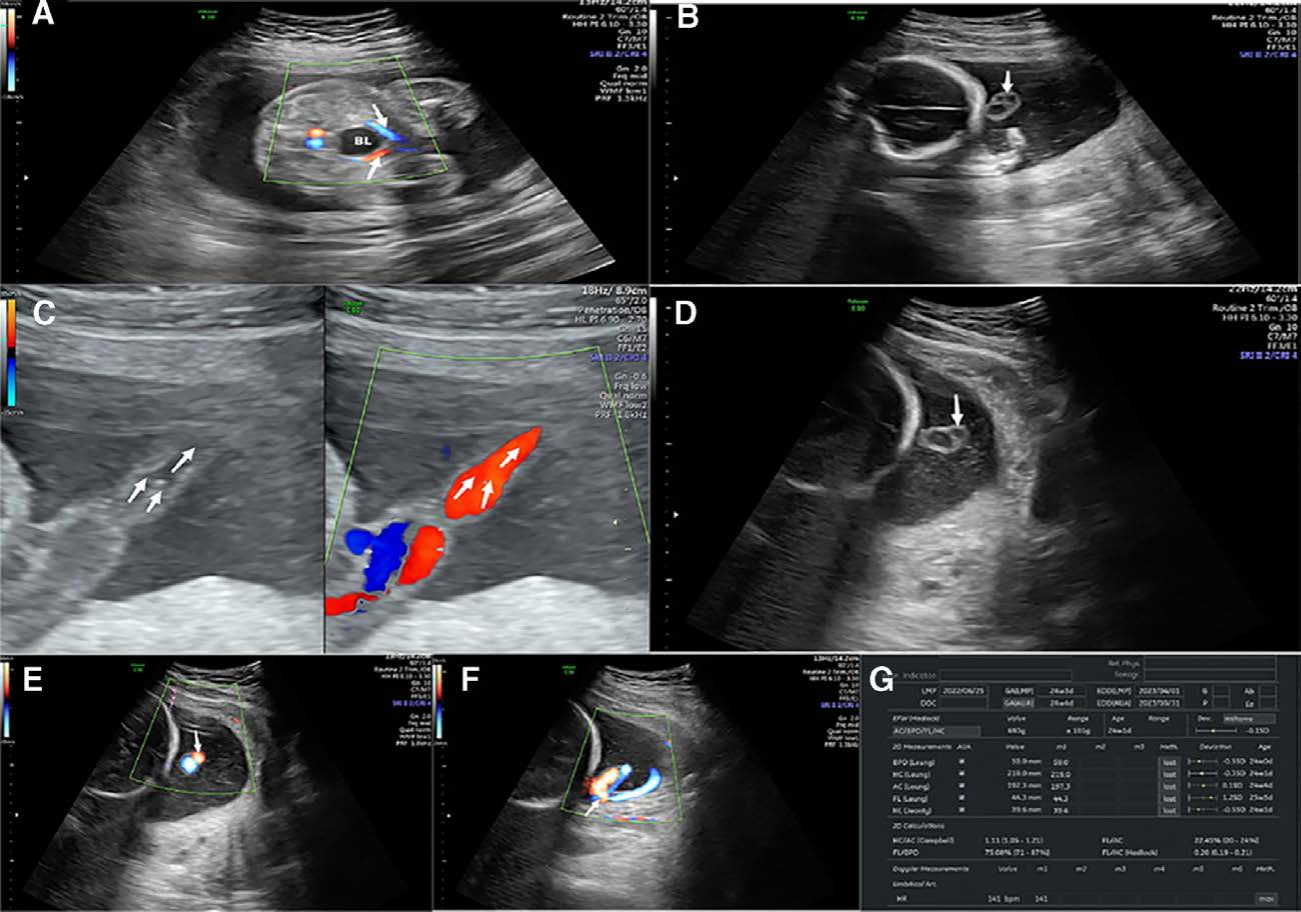
(A) Two umbilical arteries derived from the 2 arteries of the urinary bladder. (B) Normal bilateral umbilical arteries are shown in the free segment. (C) Two umbilical arteries converging into a single umbilical artery. (D) A single umbilical artery in 2 dimension. (E) A single umbilical artery shown on CDFI. (F) The umbilical cord was inserted into the amniotic sac. (G) Table of fetal biological measurements. CDFI = Color Doppler Flow Imaging.

At 24 weeks + 4 days gestation, ultrasound revealed the following umbilical artery blood flow parameters: systolic/diastolic velocity ratio = 3.9, pulsatility index = 1.28, resistance index = 0.75, heart rate = 139 beats/min, and rhythmicity. The maximum depth diameter of the amniotic fluid was 46 mm. The umbilical artery was found on both sides of the fetal bladder (Fig. 2A), with inner diameters of approximately 2.8 and 3.0 mm, respectively. Tracking and scanning from the intra-abdominal segment to the extra-abdominal segment revealed that 2 umbilical arteries converged into a single umbilical artery at approximately 40 mm from the fetal abdominal wall outlet (see Fig. 2B–E); the artery was inserted into the placental margin below the free fetal membrane along the umbilical vein. At a distance of approximately 30 mm, the artery extended from the placental margin into the placental parenchyma (Fig. 2F). The fetal biparietal diameter was 59 mm, the head circumference was 219 mm, the abdominal circumference was 197 mm, the humeral length was 39 mm, and the femoral length was 44 mm (estimated gestational age: 24 weeks + 1 day; Fig. 2G). At 31 weeks and 3 days gestation, emergency ultrasound revealed a biparietal diameter of 82 mm, a head circumference of 301 mm, an abdominal circumference of 308 mm, and a femoral length of 62 mm. The structure of the umbilical artery was consistent with the examination at 24 weeks gestation, and the umbilical artery blood flow parameters were as follows: systolic/diastolic velocity ratio = 3.0, heart rate = 146 beats/min, and rhythmicity. There was almost no amniotic fluid (premature rupture of the amniotic membrane).

## 3. Final diagnosis

**Figure 3.**
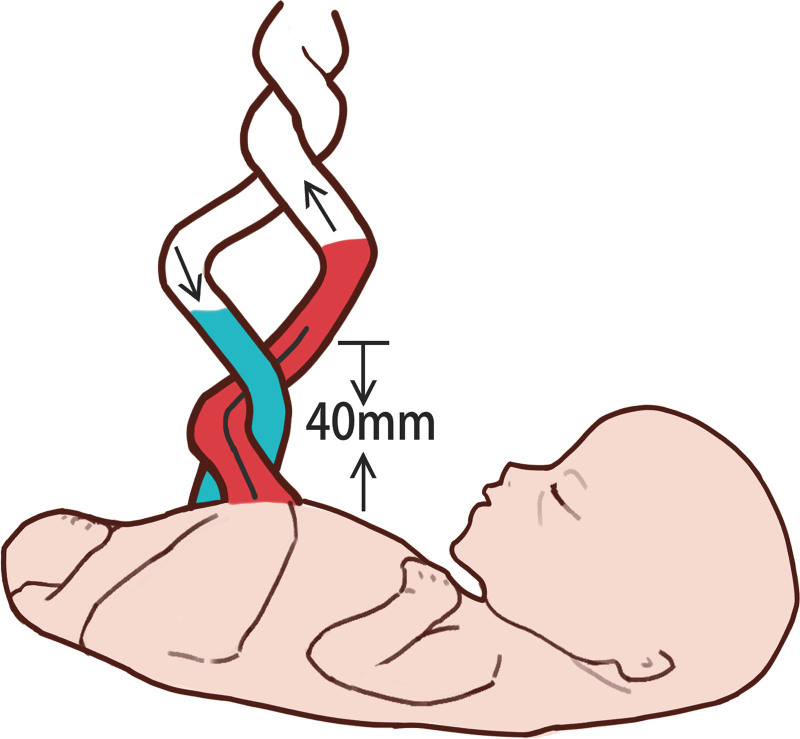
Schematic diagram of the location and blood flow direction of umbilical artery lesions.

Umbilical artery malformation (the confluence of dual umbilical arteries in the intra-abdominal segment and dual umbilical arteries in the free segment into a single umbilical artery, as shown in Fig. 3) was diagnosed.

## 4. Treatment

Two days after hospitalization for anti-inflammatory and fetal lung maturation treatment (2 g ampicillin and 250 mg erythromycin every 6 hours for intravenous infusion for 48 hours; muscle injection of dexamethasone 5 mg/12 h/time, a total of 4 times; 4 g magnesium sulfate intravenous infusion), a cesarean section was performed. The newborn was admitted to the pediatric intensive care unit, and the mother was admitted to the obstetrics department for anti-inflammatory, symptomatic, and supportive treatment.

## 5. Outcome and follow-up

After 2 days of clinical anti-inflammatory and fetal lung maturation treatment, a cesarean section was performed, and a female infant with a weight of 1620 g was delivered. The Apgar score of the infant in the first minute and 5 minutes was 9. She was admitted to the pediatric intensive care unit for observation and treatment. After 1 week of anti-inflammatory, symptomatic, and supportive treatment, the condition of both the newborn and mother improved, and they were discharged.

After 1 month, 6 months, and 1 year of follow-up after birth, the growth and development of the infant (height, weight) were significantly lower than those of her peers, and her responses to sound and light were slightly delayed.

## 6. Discussion

The umbilical cord is a special blood vessel during fetal development that serves as the only channel connecting the placenta and the fetus.^[1]^ Detection of the umbilical artery by ultrasonography can be used for intrauterine fetal monitoring and clinical guidance. Abnormal umbilical cord structure and Doppler parameters are closely related to many conditions, such as fetal chromosomal abnormalities, intrauterine infection, growth restriction, and intrauterine hypoxia.^[2–6]^

In this case, prenatal ultrasound examination at our hospital during mid-pregnancy (24 + 3 days gestation) revealed that the intra-abdominal segment had dual umbilical arteries, and the free segment had dual umbilical arteries converging into a single umbilical artery. Fetal growth and development were basically consistent with gestational age. Emergency ultrasound at 31 weeks and 3 days gestation due to premature membrane rupture revealed velar insertion of the umbilical cord and umbilical artery malformation, as observed during mid-pregnancy. The umbilical artery blood flow parameters were within the normal range, with no apparent signs of hypoxia. Ultrasound revealed that the dual umbilical arteries in the intra-abdominal segment and the free segment converged into a single umbilical artery. This is different from the physiological phenomenon of multiple umbilical artery branches converging or fusing near the placental entrance to form 1 umbilical artery.^[7,8]^ The variation position of the umbilical artery in physiological phenomena is closer to the placental side (within 30 mm of the placenta), and the number of umbilical artery vessels from the fetal side to the placental direction changes to 2 umbilical arteries branching out into multiple umbilical arteries, with a high variation rate. The rate of variation is more than 90% in all reports in the literature,^[7,8]^ and no obvious abnormalities were found in the fetus. The lesion of the umbilical artery in this patient was near the fetal side, approximately 40 mm away from the outlet of the fetal abdominal wall. The change in the number of umbilical artery vessels from the fetal side to the placental direction might be related to the fusion of 2 umbilical arteries, forming 1 umbilical artery. The anatomical location of the umbilical artery lesion in this patient differed from the physiological variation in the location, direction of vascular change, and hemodynamics reported in the literature. If only this section was examined, a missed diagnosis could occur, as typical bilateral umbilical arteries were displayed (Fig. 2A and B). It would be easy to misdiagnose this segment as a single umbilical artery if only this segment was scanned (Fig. 2D and E). Only by tracing and scanning the entire length of the umbilical artery (Fig. 2C) could the present case involving the convergence of 2 umbilical arteries into 1 artery be accurately diagnosed.

The pathogenesis and clinical significance of a single umbilical artery are entirely different from those of a single umbilical artery formed by the convergence of 2 umbilical arteries. Normally, 2 umbilical arteries and 1 umbilical vein are present in the umbilical cord, of which the 2 umbilical arteries are derived from the 2 arteries of the urinary bladder, and the umbilical vein is derived from the undegenerated urinary bladder vein on the left side. The etiology of a single umbilical artery involves early embryonic development wherein a solitary urinary bladder artery forms, while the other urinary bladder artery becomes occluded, leading to the formation of a single umbilical artery. Alternatively, one of the umbilical arteries may undergo segmental or complete occlusion due to diverse factors during embryogenesis, resulting in the formation of a single umbilical artery or a partially segmented single umbilical artery. However, the partially segmented single umbilical artery formed in this situation lacks a pathological basis for communication with the other umbilical artery at the occlusion end. Color Doppler ultrasound shows no or minimal bidirectional blood flow of the occluded umbilical artery.^[9,10]^ The prognosis of a single umbilical artery is mainly related to fetal chromosomal abnormalities, growth retardation, and intrauterine hypoxia.^[11–13]^ The pathogenesis of the formation of a single umbilical artery by the convergence of 2 umbilical arteries may be similar to that of bacterial and viral infections of tissue organs,^[8,14–18]^ for example, tissue cells and phagocytes of the mononuclear macrophage system phagocytize bacteria and viruses attached to the inner wall of the umbilical artery, leading to local epithelioid or granulomatous inflammation, marked infiltration and proliferation of tissue cells, and destruction of the normal structure of the affected umbilical artery wall, resulting in local rupture of the umbilical artery wall. Blood flows from 1 umbilical artery through the rupture into the other artery, and the distal umbilical artery is occluded due to infection and adhesion without blood flow filling. Accordingly, the merging of 2 umbilical arteries near the fetal side was primarily related to intrauterine infection. Due to intrauterine infection, the 2 umbilical arteries merged into 1, resulting in insufficient nutritional exchange between the mother and fetus. As a result of infection, the amniotic membrane becomes lesioned, and the membrane ultimately ruptures, resulting in premature birth.

The pregnant woman did not undergo Toxoplasma, Others, Rubellavirus, Cytomegalovirus, Herpesvirus, thyroid function, or biochemical tests or take omeprazole before or during the early stages of pregnancy. She only came to our hospital for prenatal ultrasound screening and fetal echocardiography at 24 weeks and 4 days gestation, and umbilical artery abnormalities could not be detected early.

## 7. Conclusion

In summary, prenatal ultrasound scanning of the entire umbilical artery can provide early indications for diagnosis, and fetuses may have an intrauterine infection caused by bacteria, viruses, or other pathogens. Ultrasound examination has the advantages of being noninvasive and cost-effective and providing real-time dynamics. Early detection of umbilical cord structure abnormalities can aid early warning of the risk of intrauterine infection, enable timely and effective treatment, and improve fetal prognosis.

## Author contributions

**Conceptualization:** Zhihui Liu, Chunfang Yang, Lihua Qiu.

**Methodology:** Zhihui Liu, Chunfang Yang, Xiaolu Yang, Lihua Qiu.

**Supervision:** Zhihui Liu.

**Writing – review & editing:** Zhihui Liu, Xiaolu Yang, Lihua Qiu.

**Investigation:** Chunfang Yang, Xiaolu Yang.

**Writing – original draft:** Chunfang Yang.
